# Monocyte Heterogeneity: Consequences for Monocyte-Derived Immune Cells

**DOI:** 10.1155/2016/1475435

**Published:** 2016-07-11

**Authors:** Sara Sprangers, Teun J. de Vries, Vincent Everts

**Affiliations:** ^1^Department of Oral Cell Biology and Functional Anatomy, Academic Centre for Dentistry Amsterdam (ACTA), University of Amsterdam and VU University Amsterdam, MOVE Research Institute Amsterdam, Gustav Mahlerlaan 3004, 1081 LA Amsterdam, Netherlands; ^2^Department of Periodontology, Academic Centre for Dentistry Amsterdam (ACTA), University of Amsterdam and VU University Amsterdam, MOVE Research Institute Amsterdam, Gustav Mahlerlaan 3004, 1081 LA Amsterdam, Netherlands

## Abstract

Blood monocytes are precursors of dendritic cells, macrophages, and osteoclasts. They are a heterogeneous cell population with differences in size, phenotype, and function. Although monocytes maintain several tissue-specific populations of immune cells in homeostasis, their contribution to populations of dendritic cells, macrophages, and osteoclasts is significantly increased in inflammation. Identification of a growing number of functionally different subsets of cells within populations of monocyte-derived immune cells has recently put monocyte heterogeneity into sharp focus. Here, we summarize recent findings in monocyte heterogeneity and their differentiation into dendritic cells, macrophages, and osteoclasts. We also discuss these advances in the context of the formation of functionally different monocyte-derived subsets of dendritic cells, macrophages, and osteoclasts.

## 1. Monocyte Phenotypical and Functional Heterogeneity

Monocytes are circulating leukocytes that are key players in tissue homeostasis and immunity. They are formed in the bone marrow and continuously enter the blood circulation, where they constitute 4% of the total leukocyte population in mice and 10% in humans [1]. In human peripheral blood, three functionally different subsets of monocytes have been identified and characterized based on their expression of surface markers CD14 and CD16 [2]. The major monocyte subset, accounting for approximately 90% of the total monocyte population, expresses high levels of CD14 and no CD16 (CD14^++^CD16^−^), and these cells are referred to as classical monocytes. Monocytes expressing CD16 can be further divided into two distinct subpopulations: intermediate monocytes that express relatively high levels of CD14 and some CD16 (CD14^+(+)^CD16^+^) and nonclassical monocytes that express low levels of CD14 and high levels of CD16 (CD14^+^CD16^++^) [3]. Analogously, mouse monocytes can be separated into two functionally different subsets based on their expression of Ly6C, CCR2, and CX_3_CR1. The Ly6C^+^CCR2^high^CX_3_CR1^low^ subset is equivalent to human classical and intermediate monocytes, whereas the Ly6C^−^CCR2^low^CX_3_CR1^high^ subset is represented by nonclassical monocytes in humans [4, 5] (Table 1). Considering the strong evidence for comparable systems, murine monocyte subsets will be referred to as their human classical/intermediate or nonclassical counterparts from now on in this review.

Monocytes represent accessory cells that can link inflammatory conditions to the adaptive immune response. Although the monocyte subsets share several common features, distinct functions have been attributed to the classical, intermediate, and nonclassical monocytes. During injury or inflammation, classical monocytes are rapidly recruited to invade the inflamed tissue and contribute to immunological responses, such as recognizing and removing microorganisms and dying cells [6]. Intermediate monocytes are recruited at a later stage of inflammation, and they are mainly associated with antigen presentation, high secretion of proinflammatory cytokines and chemokines, wound healing, and parasite recognition [7]. Nonclassical monocytes display a patrolling behavior and constantly survey the endothelium as part of the innate local surveillance [8].

**Figure 1:**
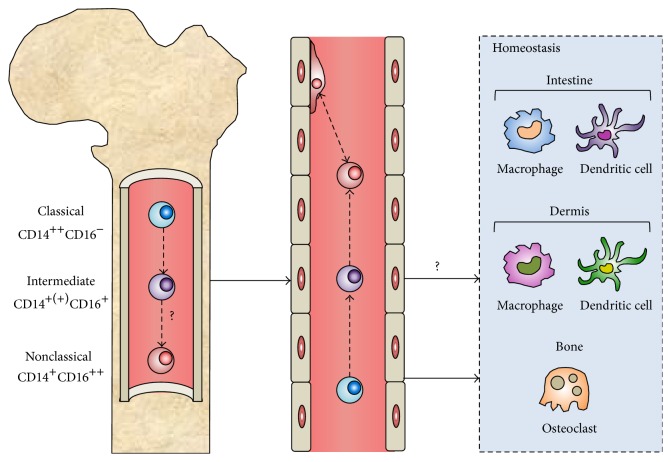
The origin and differentiation of peripheral blood monocytes. Monocytes are generated from hematopoietic stem cells in the bone marrow (left) and enter the blood stream (middle) in response to different microenvironmental cues. In homeostasis, classical monocytes are continuously recruited to populate DC and macrophage levels in the intestine and dermis and bone-degrading osteoclasts at the bone surface. It remains unknown whether intermediate monocytes contribute to populations of monocyte-derived immune cells in homeostasis. Nonclassical monocytes patrol the endothelium and do not contribute to the maintenance of populations of mature immune cells in physiology.

Although the monocyte subsets are functionally different, hierarchical clustering and gene-expression profiling have shown that the subsets represent stages in a developmental sequence, with classical monocytes differentiating into intermediate and nonclassical monocytes [9, 10]. One of the best known functions of monocytes is, however, as a systemic reservoir of precursor cells for the renewal of several populations of tissue macrophages, dendritic cells (DCs), and osteoclasts [11, 12]. In the steady-state, the precursor function is primarily associated with classical monocytes, and whether intermediate and nonclassical monocytes can function as precursors for these immune cells in homeostasis has remained more elusive (Figure 1).

**Figure 2:**
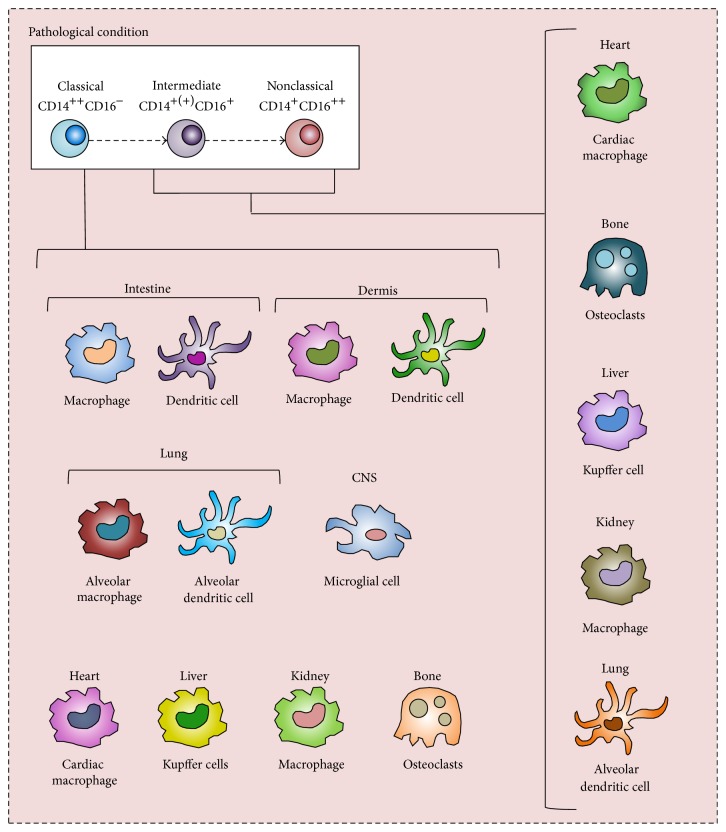
Proposed increased recruitment and differentiation of monocytes during inflammation. The contribution of monocytes to populations of mature immune cells is dramatically increased in various inflammatory conditions. Populations of immune cells normally not maintained by monocyte influx, such as populations of DCs and macrophages in the lungs, CSN, heart, liver, and kidney, are being provided by monocyte-derived counterparts during inflammation. Intermediate and nonclassical monocytes differentiate into immune cells with features distinctly different from the ones generated from classical monocytes during inflammation.

Recent evidence indicates that the renewal of tissue macrophages and DCs in the steady-state hardly relies on the recruitment of monocytes but that these populations are rather maintained by longevity and local proliferation [13]. In contrast, the contribution of monocytes as precursors is well documented during inflammation and, as a result, includes populations of immune cells normally not maintained by monocyte recruitment, such as populations of osteoclast in bone [14], macrophages in the heart [15], kidney [16], and liver [17], and DCs in the lungs [18] (see Figure 2). Inflammation favors an expansion of CD16-expressing monocytes [19], resulting in an increased contribution of intermediate and nonclassical monocytes to populations of tissue-resident immune cells. In this way, the CD16-expressing monocytes contribute to the shaping of these immune cell populations during inflammation. The role of precursor heterogeneity in the generation of immune cells during inflammation has long incited immunological research, and new understandings have put the monocyte heterogeneity into sharp focus. This raises the question of the role of monocyte heterogeneity in the development and function of mature immune cells during inflammation. In this review, we outline and evaluate the discoveries that underlie these advances in our understanding of monocyte heterogeneity and its role in the shaping of monocyte-derived populations of macrophages, DCs, and osteoclasts in homeostasis and inflammation.

## 2. Recruitment and Differentiation of Monocyte Subsets during Distinct Stages of Inflammation

**Figure 3:**
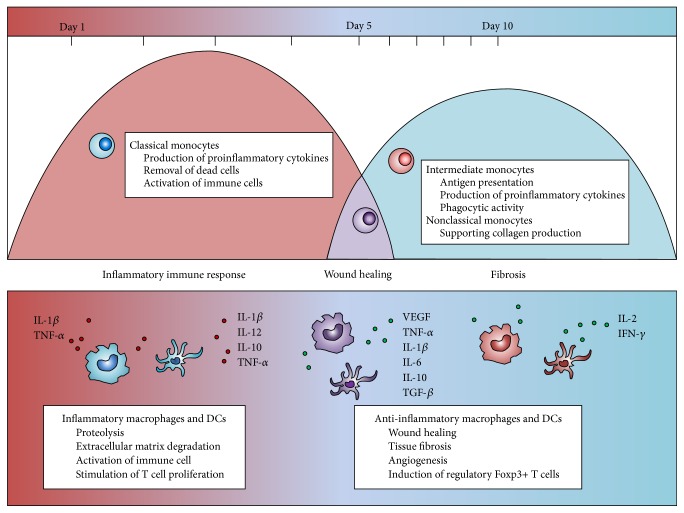
Time course of human monocyte subsets recruitment and their differentiation into macrophages and DCs during inflammation. The monocyte subsets are sequentially recruited to a site of inflammation. Their subsequent differentiation into distinct different macrophages and DCs is taking place locally and is schematically depicted above, together with their specific contributions to the resolution of the inflammation.

Monocytes are recruited sequentially to sites of inflammation as part of the host-protective immune response. In response to natural killer (NK) cell-produced interferon (IFN-*γ*), the monocytes locally differentiate into inflammatory macrophages and DCs [20] and efficiently replace the resident mononuclear phagocytes [21]. Trafficking of the monocyte subset is controlled by different mechanisms and at least two sequential phases of monocyte recruitment to sites of inflammation have been identified [22] (Figure 3). Following a myocardial infarction, classical monocytes are recruited within the first few hours, and their egression from the bone marrow is in principle controlled by the chemokine receptor CCR2 and its ligands CCL2 (or MCP-1) and CCL7 (or MCP-3) [23]. The recruited classical monocytes arrive in a highly inflammatory milieu where they exert an immediate and potent immune response by producing high levels of proinflammatory cytokines, such as interleukin IL-1*β* and TNF-*α*. In addition, they locally digest extracellular matrix and dead cells [24] and produce IL-18 to activate NK cells [25], thereby playing an important role in the progression of the immune response. A prolonged immune response from classical monocytes can contribute to tissue damage and initiate inflammatory cascades, as well as drive autoimmunity [26, 27].

Some days later, when the acute inflammation resolves into a cardiac wound, the presence of classical monocytes diminishes and they are subsequently replaced by intermediate and nonclassical monocytes. In contrast to classical monocytes, CD16-expressing monocytes express low levels of CCR2 and rely on migration signals mediated by chemokine receptor CX_3_CR1 and its ligand CX_3_CL1 [28]. The nonclassical monocytes accumulate in the damaged tissue and contribute to angiogenesis and fibrosis [29, 30]. By secreting anti-inflammatory cytokines, such as IL-10 and transforming growth factor TGF-*β*, the CD16-expressing monocytes/macrophages counteract the tissue damage caused by an aggressive immune response from classical monocytes/macrophages [31].

The distinct recruitment of the three monocyte subsets was recently also observed when studying an infected kidney mouse model [16], where classical monocytes/macrophages were observed to appear rapidly after infection. Here, they expressed genes associated with immune response and monocyte/macrophage differentiation. Intermediate monocytes/macrophages arrived later and expressed genes associated with wound healing and released vascular endothelial growth factor and TGF-*β*, supporting angiogenesis and collagen production. The nonclassical monocyte/macrophage population peaked 10 days after the kidney infection and expressed genes associated with fibrosis [16]. Similar observations have been made in patients with chronic inflammatory and fibrotic liver diseases, where intermediate monocytes accumulated in the inflamed liver as a consequence of enhanced recruitment of these monocytes from the circulation and local differentiation of classical monocytes in response to inflammatory factors [17]. The same study concluded that these intermediate monocytes expressed both early macrophage and DC markers and were associated with increased phagocytic activity, antigen presentation, and secretion of proinflammatory cytokines (such as tumor necrosis factor TNF-*α*, IL-6, and IL-1*β*) and different growth factors consistent with a role in wound healing [17, 32]. Thus, observations in both murine disease models and human patients suggest that the delayed recruitment of intermediate and nonclassical monocyte subsets and their subsequent differentiation into macrophages and DCs is a conserved mechanism that reflects a host-driven response to limit possible tissue damage caused by strong immune responses from classical monocytes/macrophages. It should be noted, however, that the fate of differentiated monocytes after resolution of inflammation remains as a subject of debate, although it has been suggested that they are able to undergo* in situ* phenotype conversation to become tissue-resident macrophages [9].

## 3. Inflammation Enhances Monocyte Contribution to the Tissue-Resident Cell Populations 

Monocytes can function as precursors of DCs, macrophages, and osteoclasts. However, the fact that monocytes are the immediate upstream precursors of these specialized cell populations is a dogma that only recently was refined with the usage of sophisticated fate-mapping techniques and different* in vivo* disease models [9, 33]. Instead of depending on monocyte recruitment, several tissue-resident macrophage and DC populations rather appear to be maintained through longevity and local proliferation of precursors seeded during the embryonic development [13]. Yet, depletion of tissue-resident cell populations has demonstrated that circulating precursors in the blood can replenish numerous populations of specialized macrophages and DCs [34, 35], supporting the idea of blood monocytes as a circulating precursor reservoir that can be exploited on demand. Although classical monocytes are contributing to some populations of tissue-resident DCs, macrophages, and osteoclasts in the steady-state, monocyte recruitment is strongly increased during inflammation and the affected distribution of monocytes, favoring an expansion of CD16-expressing monocytes [36], has great impact on the formation of monocyte-derived immune cells during inflammation.

### 3.1. Monocyte-Derived Dendritic Cells

Dendritic cells (DCs) are professional antigen-presenting cells and key regulators of innate and adaptive immune responses. A number of positive DC lineage markers have been identified that separates DCs into either “classical” or “plasmacytoid” DCs [37]. The latter are not derived from circulating monocytes and are therefore not discussed further in this review. Monocytes cultured in the presence of granulocyte macrophage-colony stimulating factor (GM-CSF) and IL-4 generate immature DC that differentiate further into mature DCs by TNF-*α* stimulus [38, 39]. Within the total population of classical DCs, several distinct subpopulations have been identified, each possessing distinct phenotypical and functional features [40–42]. Although DCs primarily are generated from pre-DCs in the circulation [43], selected DC populations in the dermis and the intestine are continuously repopulated by recruited classical monocytes [34, 44]. Evidence supporting a role for CD16-expressing monocytes in the replenishment of DC populations in steady-state is lacking, and it is possible that the patrolling nonclassical monocytes leave the blood vessels and function as DC precursors exclusively in response to inflammatory stimuli [45]. During inflammation, monocyte differentiation into DCs is not restricted to the skin or intestine but includes peripheral tissues normally not maintained by monocyte input [46] (Figure 2).

The monocyte-derived DCs during inflammation have unique features, distinctly different from tissue-resident DCs generated during steady-state conditions.* In vivo* transfer experiments have shown that injected monocytes migrate to inflammatory sites and differentiate into DCs in various models of inflammation, including rheumatoid arthritis [47] and Dengue virus infection [48]. As part of the innate immune system, monocyte-derived DCs during inflammation secrete high amounts of the anti-inflammatory cytokine IL-10 and engulf apoptotic erythroid cells. Accordingly, blocking differentiation of monocytes into DCs results in tissue damage due to severe and prolonged inflammation, cytotoxic T cell activity, and shortened host survival expectancy [49]. Monocyte-derived DCs have been suggested to contribute to the regulatory control of immune responses [50], and they produce large amounts of proinflammatory cytokines and enhance Th2 cell-mediated immunity in the lungs [51]. The specific contributions of classical, intermediate, and nonclassical monocytes to DC populations during inflammation were recently investigated in patients suffering from end stage renal disease, where chronic inflammation and dramatically increased numbers of circulating nonclassical monocytes were associated with an increased generation of DCs [52]. The specific contribution of the monocyte subsets to populations of DCs during inflammation has long been a topic of debate, and it has been reported that functional differences exist between DCs generated from the different monocyte subsets [53]. These differences include more potent immune responses from DCs derived from classical monocytes and better immune tolerance from DCs generated from nonclassical monocytes [54]. Similarly, it has been reported that classical monocytes selectively repopulate populations of CD103^+^ DCs, whereas nonclassical monocytes differentiate into populations of CD11b^high^ DCs in the lungs [55]. More recently, these findings were supported by similar observations reported in patients with tuberculosis. Patients with tuberculosis have increased numbers of both intermediate and nonclassical monocytes in the circulation [56], and the CD16-expressing monocytes in these patients differentiate into DCs with poor mycobacterial antigen-presenting capacity [18]. This is explained by the observation that stimulated CD16-expressing monocytes differentiate into alternative DCs with poor antigen-presenting function, expressing no CD1a and low levels of DC-SIGN on their plasma membrane [18]. After LPS stimulation, these inflammatory DCs produce large amounts of IL-2 and IFN-*γ*, further driving the differentiation of monocytes into inflammatory mature immune cells [57]. Classical monocytes, on the other hand, generate functional CD1a^+^DC-SIGN^high^ DCs that efficiently stimulate T cell proliferation and secrete high amounts of IL-12, IL-1*β*, IL-10, and TNF-*α* upon* Mycobacterium tuberculosis* infection or LPS stimulation [18].

The increased presence of circulating CD16-expressing monocytes during inflammation appears to play a critical role in the development of DCs also in other pathological conditions. For example, in sepsis—a systemic inflammatory response syndrome that occurs during infection—an expansion of intermediate monocytes has been detected in the blood circulation [6]. Monocytes derived from sepsis patients preferably differentiate into alternative CD1a^−^ DCs (similar to the DCs derived from CD16-expressing monocytes in patients with tuberculosis discussed above) [58]. These alternative DCs have an increased capacity to induce regulatory Foxp3+ T cells, as compared with monocytes derived from healthy controls with a higher distribution of classical monocytes [58]. Thus, a growing body of circumstantial evidence suggests that the monocyte subsets give rise to functionally distinct DCs during inflammation and that the enhanced presence of circulating intermediate and nonclassical monocytes shapes populations of DCs during pathological conditions.

### 3.2. Monocyte-Derived Macrophages

Macrophages are exquisitely adapted to their local environment and acquire organ-specific functionalities as part of their role in the maintenance of tissue homeostasis. Development, differentiation, proliferation, and function of macrophages are regulated by the growth factor colony stimulating factor CSF-1 and IL-34 [59]. Macrophages belong to a heterogeneous cell population, with several phenotypically and functionally distinct subsets [58]. Most macrophage populations are established prior to birth and maintain themselves by longevity and local proliferation, rather than monocyte recruitment. These macrophage populations include microglia in the central nervous system, Kupffer cells in the liver, peritoneal macrophages, and splenic macrophages [9, 13]. Microglia was early shown to originate from embryonic progenitors [60], and more recent research has identified the microglia precursors as primitive macrophages in the yolk sac [61].

Yet, in other tissues, including the intestine [62] and the dermis [63], classical monocytes are continuously recruited to maintain the local macrophage populations in homeostasis. In addition, monocyte-derived cardiac macrophages appear to replace macrophages seeded during the embryonic development throughout the life span of an individual [64]. It has been reported that monocyte-derived macrophages, similar to monocyte-derived DCs, are functionally different from their tissue-resident counterparts. Monocyte-derived macrophages in the intestine express higher levels of CXC_3_R1 [65], induce differentiation of Foxp3+ T cells from naïve CD4^+^ T cells [66], and are required for induction of Th17 cells and antigen-specific responses [65]. Whether CD16-expressing monocytes also contribute to macrophage populations in steady-state is unknown, and although early reports indicated that nonclassical monocytes differentiate into alveolar macrophages in homeostasis [67, 68], more recent research indicates that alveolar macrophages are in fact derived from fetal monocytes with minimal contribution of circulating blood monocytes [69].

Although classical monocytes appear to be the primary precursors of selected populations of macrophage during steady-state, the recruitment of all monocyte subsets during inflammation is strongly increased. Inflammatory insults result in recruitment of monocytes to populations of tissue-resident macrophages that normally are maintained independently of monocyte influx, such as macrophages in the heart [70], in the ischemia brain tissue [71], and in the inflamed liver tissue [31] (Figure 2). The monocyte heterogeneity plays an important role in the generation of functionally distinct macrophages and the monocyte subsets appear to function as macrophage precursors in different pathological conditions. For example, infection with helminth parasites* Schistosoma mansoni* and* Heligmosomoides polygyrus* results in rapid invasion of classical monocytes into the adult murine heart, where they drive inflammation and generate oxidative stress [72]. These classical monocytes subsequently differentiate into macrophages with limited capacity to promote tissue repair [73]. However, in the absence of parasite challenge, such as during cardiac pressure overload, preferential recruitment and accumulation of nonclassical monocytes/macrophages in the cardiac tissue have been observed [15]. Similar to the selective recruitment of monocytes discussed above in Section 2, the sequential differentiation of the monocyte subsets into macrophages in response to myocardial challenges is likely due to the individual features of the different monocytes/macrophages.

Macrophages derived from the different monocyte subsets have been shown to maintain some of the properties of their progenitors. For example, macrophages derived from classical monocytes express higher levels of CD14 on their surface compared to macrophages derived from nonclassical monocytes when cultured* in vitro* [74]. While macrophages from classical monocytes exhibit phagocytic, proteolytic, and inflammatory functions, macrophages derived from CD16-expressing monocytes promote healing of the cardiac tissue by angiogenesis and deposition of collagen [75]. The functional differences between macrophages derived from classical and CD16-expressing monocytes have given rise to the idea that classical monocytes differentiate into cardiac M1 macrophages, whereas CD16-expressing monocytes become M2 macrophages [76]. This, however, still needs to be confirmed. In either way, the selective recruitment of specific monocyte subsets is context dependent and based on the nature of the challenge. Thus, the sequentially recruited monocyte subsets during inflammation differentiate locally into macrophages with distinct capacities to drive inflammatory responses or promote tissue repair.

### 3.3. Monocyte-Derived Osteoclasts

Osteoclasts comprise a subset of specialized macrophages that arise from fusion of monocytes in the presence of the cytokines macrophage-colony stimulating factor (M-CSF) and receptor activator of NF-*κ*B ligand (RANKL). These cells are uniquely capable of resorbing mineralized tissue, like bone, by binding tightly to the surface and by degrading the different matrix components by secreting acid followed by a cocktail of different proteolytic enzymes. Although not considered traditional immune cells, a growing body of evidence suggests that osteoclasts contribute to inflammation and immune responses via the release of cytokines and via antigen presentation [77]. Functional differences between subsets of osteoclasts have been reported in homeostasis and include differences in size and proteolytic enzymes used for bone matrix digestion [78]. Although it was long assumed that monocytes are an important source of osteoclast precursors, this was not proven* in situ* until recently when fluorescently labeled monocytes were recruited from the circulation to the bone surface and differentiated locally into osteoclasts [79]. Accordingly, depletion of blood monocytes decreases osteoclastic bone degradation by limiting the homing of precursors to the bone surface [80].

Bone degradation by osteoclasts is crucial for skeletal maintenance, but increased and uncontrolled bone degradation during inflammation results is a severe pathological phenotype [81]. Due to their roles as osteoclast precursors, circulating monocytes form an excellent tool to study the early onset of inflammatory bone loss [82]. In healthy individuals, it is the classical monocytes that harbor the highest propensity to differentiate into osteoclasts [83]. Interestingly, however, Chiu et al. in 2010 reported a major shift in osteoclast precursors, from classical monocytes in healthy individuals towards an increased osteoclast formation from intermediate and nonclassical monocytes in patients with psoriatic arthritis (a chronic inflammatory arthritis characterized by severe bone erosion) [84]. The influence of an affected distribution of circulating monocytes on osteoclast formation during inflammation has also been observed when osteoclastogenesis of monocytes from patients with inflammatory bone loss has been studied in detail. Monocytes isolated from patients with Gaucher's disease form osteoclasts faster than monocytes isolated from healthy controls and the generated osteoclasts display an increased bone-resorptive capacity when compared with osteoclast derived from monocytes isolated from healthy controls [85]. Similar observations were reported for patients with rheumatoid arthritis, where osteoclasts with increased bone-resorptive capacity were generated from monocytes derived from patients with rheumatoid arthritis [86]. This indicates that it is in fact intrinsic properties of the isolated monocytes that cause the generation of distinct different osteoclasts and not altered cytokine levels in an inflammatory environment. Interestingly, in all above-mentioned conditions (psoriatic arthritis, Gaucher's disease, and rheumatoid arthritis), a selective expansion of the intermediate monocyte subset has been reported, suggesting that in particular this subset is involved in the formation of functionally distinct osteoclasts in these inflammatory conditions [6, 87, 88]. Accordingly, we recently demonstrated that in particular osteoclasts generated from intermediate monocytes expressed an increased capacity to resorb bone when they are treated with the inflammatory cytokine IL-17A [14]. Taken together, the increased numbers of CD16-expressing monocytes and in particular intermediate monocytes appear to play a critical role in the generation of osteoclasts during inflammation and can possibly serve as an explanation for the increased osteoclast-associated bone loss observed in several inflammatory disorders.

## 4. Concluding Remarks and Future Perspectives

The role of monocytes as precursors for various mature immune cells has been well established. As our understanding of monocyte heterogeneity improves, their intriguing role as precursor cells is becoming increasingly important, and targeting of specific monocyte subsets to control differentiation and function of monocyte-derived immune cells emerges as an appealing therapeutic approach. The putative role of classical, intermediate, and nonclassical monocytes as distinct precursor cells during inflammation is of particular interest for immunological research, but our knowledge is limited and several important aspects are still unknown. This is partly due to the fact that the data collected so far mainly consists of* in vitro* observations and, unfortunately, few studies have investigated the correlation between an affected precursor population and the development of unconventional downstream immune cells during inflammation. Defining the distinct differentiation fates of the monocyte subsets in different inflammatory conditions will enable more precise targeting of immune cells and provide a better understanding of the pathophysiology of inflammation.

## Figures and Tables

**Table 1:** Human monocyte subsets and their murine counterparts.

Subset	Markers	Chemokine receptors	Main functions
Human			
Classical	CD14^++^CD16^−^	CCR2^high^CX3CR1^low^	Immune response Phagocytosis
Intermediate	CD14^+(+)^CD16^+^	CCR2^low^CX3CR1^high^	Proinflammatory Wound healing
Nonclassical	CD14^+^CD16^++^	CCR2^low^CX3CR1^high^	Patrolling role Fibrosis
Mouse			
Classical/intermediate (*∗*)	Ly6C^+^CD11b^+^CD115^+^	CCR2^high^CX3CR1^low^	Proinflammatory Phagocytosis
Nonclassical	Ly6C^−^CD11b^+^CD115^+^	CCR2^low^CX3CR1^high^	Patrolling Tissue repair

(*∗*) Murine Ly6C^+^ (classical/intermediate) monocytes are sometimes further divided into Ly6C^high^ and Ly6C^intermediate^ monocytes.
